# Patients With Suicidal Patterns in the Emergency Room: A Clinical and Social Reflection

**DOI:** 10.7759/cureus.18570

**Published:** 2021-10-07

**Authors:** Rajesh K, Amogh Ananda Rao, Krishna D, Pratyaksh P Vaishnav, Sissmol Davis, Abhinov T, Devendraprasad K J, Ashutosh Suresh, Chiranth Nadig

**Affiliations:** 1 Emergency Medicine, Sri Devaraj Urs Medical College and Research Hospital, Kolar, IND; 2 Internal Medicine, Jagadguru Jayadeva Murugarajendra (JJM) Medical College, Davangere, IND

**Keywords:** suicide attempt, suicide prevention, suicide behavior, psychiatry & mental health, emergency medicine and trauma, research in emergency medicine, social psychiatry, public mental health

## Abstract

Background and objective

Emergency physicians come across a myriad of medical afflictions resulting from suicide attempts. The Indian contribution to global suicide deaths is alarming; the social construct of India is unique, and so are the problems and challenges. This study aims to describe the social background, demographic parameters and correlate the clinical profile and outcomes of all patients presenting with an attempted or completed suicide.

Materials and methods

The present study is a hospital-based prospective observational study conducted by the Department of Emergency Medicine at RL Jalappa Hospital and Research Centre, a rural tertiary-care center in Karnataka, India, from June 2020 to February 2021. After stabilizing the patient, a detailed socio-demographic history was recorded. Details of the suicide attempt and findings of the clinical examination were noted.

Results

The final study sample consisted of 89 patients who presented to the emergency room (ER). Fifty-three patients were female, and thirty-six were males; the average age of the sample was 28.4 ± 11 years. A higher proportion of men who completed pre-university education (p= 0.0005, c^2^= 11.98) or had a graduate degree (p= 0.009, c^2^= 6.71) attempted suicide. Amongst all women who attempted suicide, 73.6% (n=39) were married at the time of the event (p= 0.0006, c^2^= 11.79). Poisoning (n=59) was the most common method of attempting suicide. We also observed that it was primarily men who attempted suicide when under the influence of alcohol (p= 0.006, c^2^= 7.57). The most common reason for attempting suicide was familial disharmony, including domestic violence. A Glasgow Coma Scale (GCS) score of 9/15 and less at the time of presentation resulted in a mortality rate of 28.6%, whereas patients with a GCS score of 10 and above had a mortality rate of 6.1% (p= 0.04, c^2^= 4.14).

Discussion

Marriage appears to be less protective for Indian women than Indian men. Poisoning was the most common method of attempted suicide in our study, followed by tablet overdose. The reason for the above could be ease of access to household poisons. Insecticides have been a preferred method in the Indian population over the years. Aluminum phosphide poisoning, a common constituent of rodenticides, is associated with a high mortality rate. However, in the West, firearm-related incidents have the highest mortality. Multiple correspondence analysis (MCA) of the National Crime Records Bureau (NCRB), India, data showed that adult males succumb majorly to romantic relations, unemployment, and failure in examinations. The use of alcohol was more in the illiterate and unskilled workers; however, high school educated persons and students used alcohol intentionally to facilitate suicide. Lower Glasgow Coma Scale values are associated with higher fatality; however, some studies found that Full Outline of Unresponsiveness (FOUR) and Acute Physiology and Chronic Health Evaluation Score (APACHE) II scores are better mortality indicators.

Conclusion

Besides the presentation and GCS score, cognizance of the lethality of different methods in attempting suicide provides clues in anticipating the patient's clinical course. The social patterns of suicide must be considered while designing awareness campaigns and focused outreach programs to decrease suicides. A strict policy must be made and enforced to limit the availability of household poisons.

## Introduction

Emergency physicians come across a myriad of medical afflictions resulting from suicide attempts [1,2]. According to the 2019 WHO report, the age-standardized suicide rate in India is 1.25 per million population, against the global average of 0.9 per million population [3]. However, it is expected to witness an increase after the decriminalization of suicide in India has addressed under-reporting. Shockingly, suicide is the most common cause of death in the age group of 15-39 years, masking what could be a potential mental health epidemic [4].

The Indian contribution to global suicide deaths is an alarming share of 36.6% among women and 24.3% among men, despite making up around 18% of the global population. The social construct of India is unique, and so are the problems and challenges. There is considerable variability, owing to economic prosperity, religious beliefs, and the way of dealing with stress. It is also unfortunate that suicide is the commonest cause of death amongst the youth in India; yet again, a deviation from the global trend. Therefore, studies investigating the story behind these suicides are necessary to reduce the rates at which people attempt suicides [5-7].

This study aims to describe the social background, demographic parameters and correlate the clinical profile and outcomes of all patients presenting with an attempt or a completed suicide. Elucidation of behavioral patterns existing in society will allow timely medical and psychological intervention in high-risk groups to prevent suicides.

## Materials and methods

This is a hospital-based prospective observational study conducted by the Department of Emergency Medicine at RL Jalappa Hospital and Research Centre, a rural tertiary-care center, from June 2020 to February 2021. The Institutional Ethics Committee of Sri Devaraj Urs Medical College, Tamaka, Kolar examined and unanimously approved the study with approval number SDUMC/KLR/IEC/11/2020-21. After obtaining a clearance from the Institutional Ethics Committee, all patients presenting to the emergency room (ER) with an attempted or a completed suicide were included in the study. Non-consenting patients and cases of accidental consumption of poison were excluded. Estimation of the desired sample size could not be performed since region-specific data of attempted suicides is sparse.

After stabilizing the patient, a detailed socio-demographic history was recorded (Table 1). Details of the suicide attempt and findings of the clinical examination were noted. The assistance of the relatives/attenders was sought in some cases. The patients were then administered the Patient Health Questionnaire-9 (PHQ9) in the local language to screen for depression. Confidentiality and depersonalization of data were communicated to the patient and their family. No personal identification was documented in order to maintain the privacy of the patients.

**Table 1:** Parameters Observed in the Study AS- Attempted Suicide; H/o- History of; PHQ-9- Patient Health Questionnaire 9

SOCIODEMOGRAPHIC FACTORS	INFORMATION REGARDING THE ATTEMPTED SUICIDE (AS)	CLINICAL PARAMETERS
Age	Mode of AS	Presenting Complaint
Sex	Reason for AS	H/o Psychiatric Illness
Education	History of Previous AS	Vital Parameters
Occupation	Time and Location of AS	Glasgow Coma Scale
Family Income	Prior Communication	PHQ-9 Score
Relationship Status	Impulsive/Planned	Duration of Hospital Stay
Family type	Ascertainment of AS	Outcome

Statistical analysis was performed on Microsoft Excel for Windows 10, version 2104. The socio-demographic data and an account of the suicidal patterns are presented in the form of descriptive statistics. The chi-square test was used to test the significance of associations amongst categorical variables. The t-test, simple linear and logistic regression were also employed.

## Results

The final study sample consisted of 89 patients who presented to the ER. 53 patients were female, and 36 were males; the average age of the sample was 28.4 ± 11 years (Table 2). The difference between the average age of males (31.75 ± 10.9 years) and that of females (26.13 ± 10.2 years) was statistically significant (p = 0.01, t = 0.007).

**Table 2:** Age and sex distribution of the study sample SD: Standard Deviation

Number of Patients		Female	Male	Total	Significance
		53	36	89	p = 0.01, t = 0.007 (Significant)
Age (in years)	Average	26.13	31.75	28.4	
	SD	10.2	10.9	11	
	Maximum	65	70	70	
	Minimum	15	17	15	

**Figure 1:**
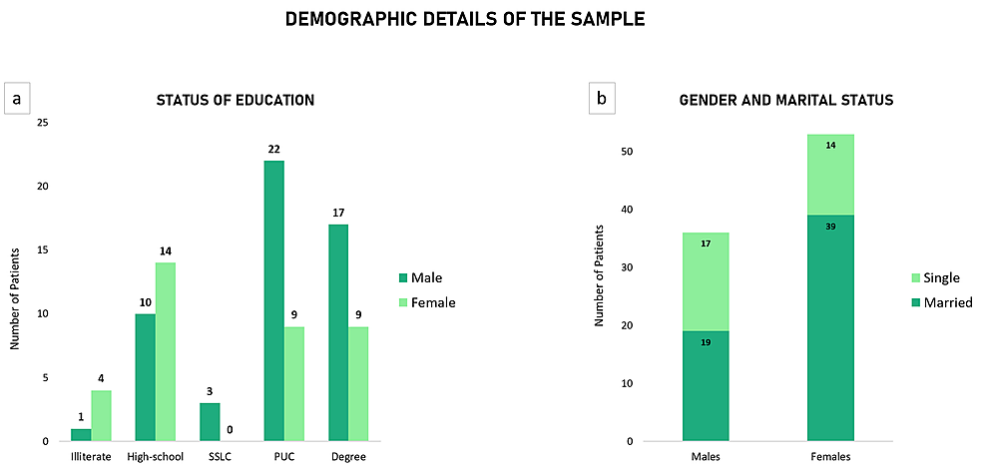
The correlation of attempted suicides with: a) Status of education; b) Gender and marital status SSLC: Secondary School Leaving Certificate, an equivalent of Class 10 (Grade 10); PUC: Pre-University College, an equivalent of Class 12 (Grade 12)

In terms of education, a higher proportion of men who completed pre-university education (p= 0.0005, $\chi$^2 ^= 11.98) or had a graduate degree (p= 0.009, $\chi$^2^= 6.71) attempted suicide (Figure 1a). Amongst all women who attempted suicide, 73.6% (n=39) were married at the time of the event (p= 0.0006, $\chi$^2 ^= 11.79). However, there was no significant difference amongst married and unmarried/widowed men (Figure 1b). The family income and composition did not have a significant impact on attempted suicides in our study.

**Figure 2:**
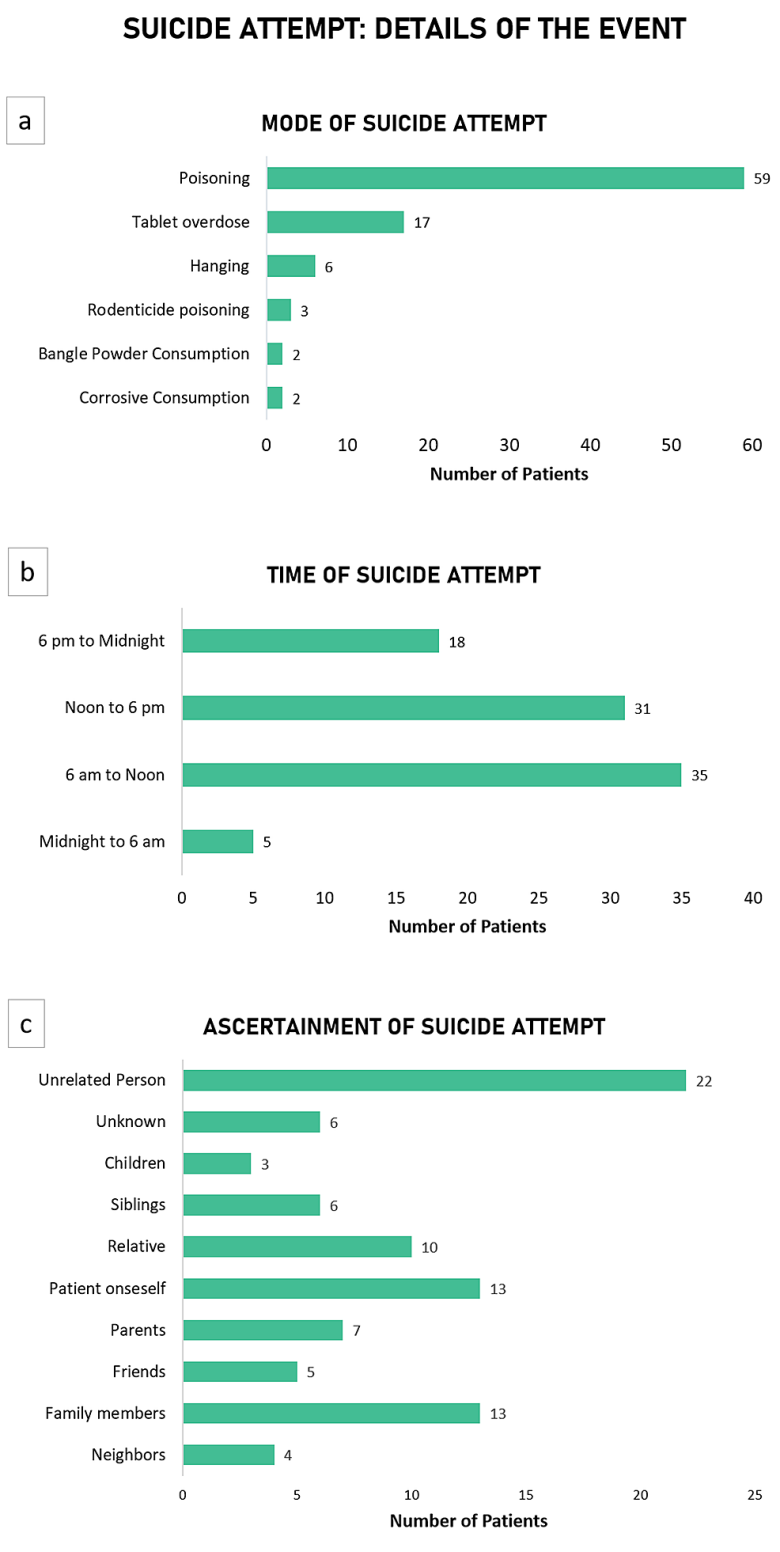
Details of suicide attempt events a) Mode of suicide attempts; b) Temporal distribution of the time of suicide attempt; and c) The ascertainment of the suicide attempt.

Poisoning (n=59) was the most common method of attempting suicide; significantly so amongst men (91.6%, n=33) in comparison with women (49.1%, n=26) (p= 0.01, $\chi$​​​​​​​^2^= 5.87). Overdosing on pharmaceuticals as a mode of attempting suicide was significantly more common amongst women compared to men (p= 0.0006, $\chi$​​​​​​​​​​​​​​^2^= 11.55) (Figure 2a). Suicide attempts mostly occurred between 6 a.m. and 12 p.m. (n=35), and between 12 p.m. and 6 p.m. (n=31) (p< 0.0001, $\chi$​​​​​​​​​​​​​​^2^= 24.93) (Figure 2b). Since the COVID-19 lockdown was in place, the locations of attempted suicide were either the patients’ residence (n=71, 79.8%) or farmland (n=16, 17.9%). In most cases, an unrelated person (n=22, 24.7%) first noticed the event of attempted suicide and informed the patients’ families. In some cases, the patients (n=13, 14.6%) themselves informed the family (Figure 2c). Education and financial status did not have a significant bearing on the suicidal method.

In the studies cases, 92% of the suicide attempts were impulsive acts, whereas 8% chalked out a plan well before the event. Three patients had a history of previous suicide attempts. It was more common amongst men to communicate prior to the impending suicide attempt (p= 0.036, $\chi$​​​​​​​​​​​​​​^2^= 4.36). We also observed that it was primarily men who attempted suicide when under the influence of alcohol (p= 0.006, $\chi$​​​​​​​​​​​​​​^2^= 7.57).

**Figure 3:**
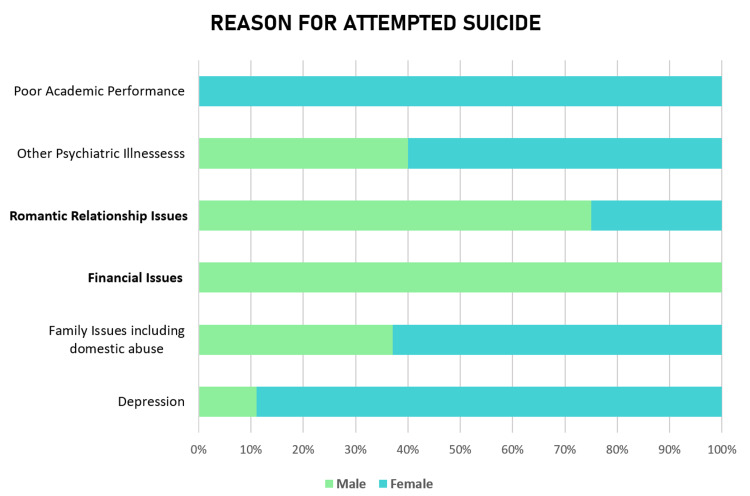
Comparison between males and females based on the reason for attempted suicide A significantly higher number of males attributed financial issues and problems in romantic relationships as the reason for attempting suicide. Previously diagnosed depression was categorized separately from other psychiatric illnesses.

The most common reason for attempting suicide was familial disharmony, including domestic violence (64% in males and 73% in females). A significantly higher proportion of males (males: 17%, females: 4%) attempted suicide due to problems and failures in romantic relationships than females (p= 0.046, $\chi$​​​​​​​​​​​​​​^2^= 3.96). Similarly, the attribution of suicide attempts to financial issues was significantly higher amongst males (p= 0.015, $\chi$​​​​​​​​​​​​​​^2^= 5.88) (Figure 3).

**Figure 4:**
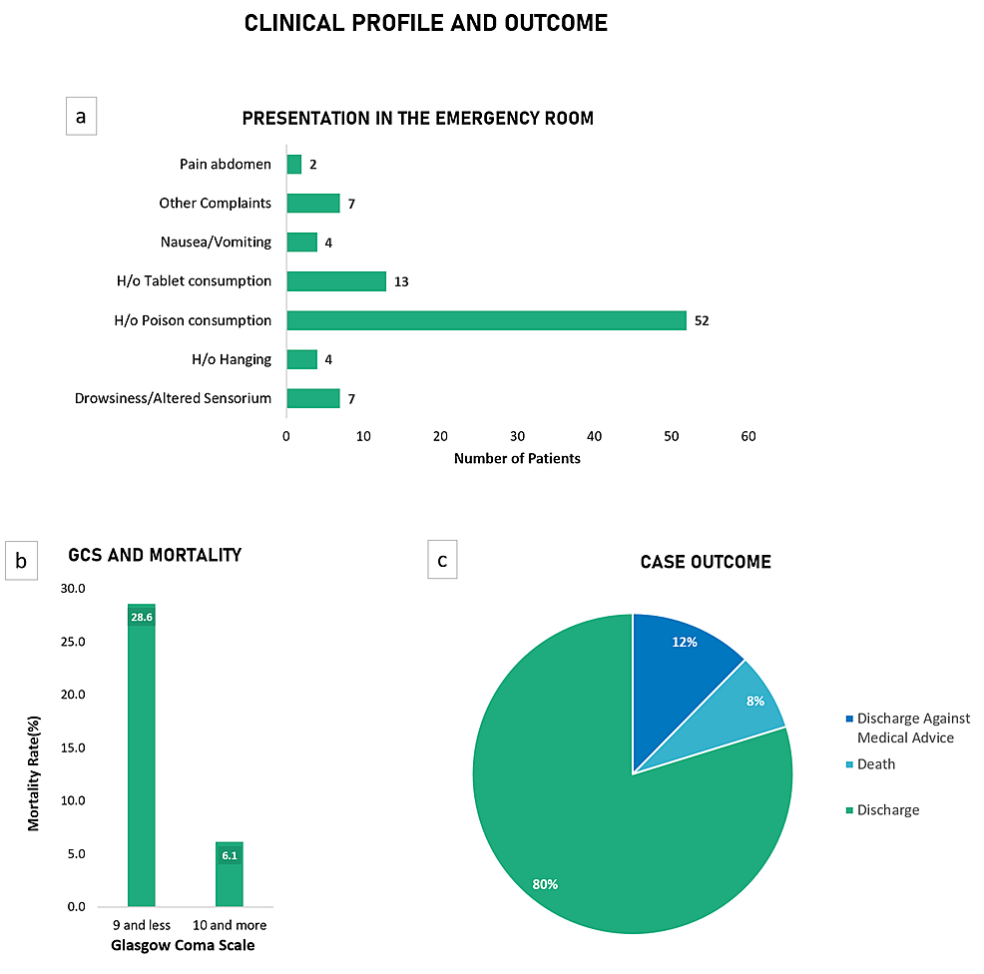
Clinical profile and outcomes of the patients presenting to the emergency room a) Presenting complaints of the patients in the ER; b) Comparison between a GCS score and mortality rates; c) Pie chart indicating the case outcomes ER: Emergency Room; GCS: Glasgow Coma Score.

The presentation of patients to the emergency room was congruent with the mode of suicide attempts (Figure 4a). A Glasgow Coma Scale (GCS) score of 9/15 and less at the time of presentation resulted in a mortality rate of 28.6%, whereas patients with a GCS score of 10 and above had a mortality rate of 6.1% (p= 0.04,$\chi$​​​​​​​​​​​​​​^2^= 4.14) (Figure 4b). The majority of patients, 80%, recovered and were discharged (Figure 4c). However, around 12% of the patients insisted on discharge before the physician’s advice. The overall mortality rate in our study was 7.8%; rodenticide consumption was the most lethal mode of attempted suicide, having a mortality rate of 66.7%.

**Figure 5:**
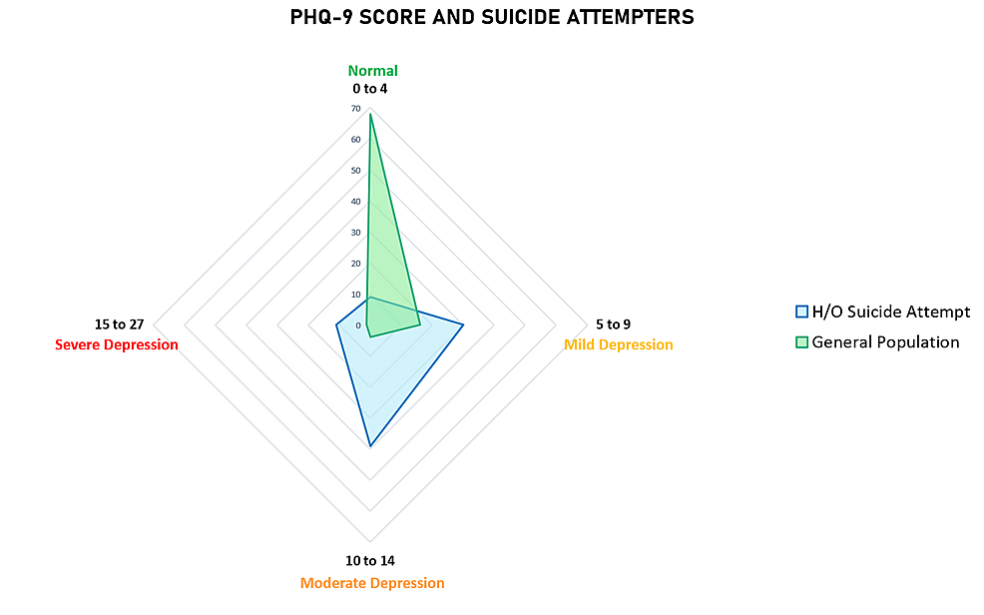
PHQ-9 score comparison between our study sample and the general population. PHQ-9: Patient Health Questionnaire; H/O: History of

In our study, 10.1% (n=9) patients had a previously established diagnosis of a psychiatric illness. However, on screening the patients with the Patient Health Questionnaire-9 (PHQ9), we found that 56.2% (n=50) patients had a score of 10 and above, reflecting moderate and severe depression. Figure 5 shows the distribution of PHQ-9 scores between our study sample and the general population.

## Discussion

The following are the significant observations made in this study: A significant proportion (73.6%) of women who attempted suicide were married at the time of the event; Poisoning was the most common method of attempting suicide, followed by tablet overdose; It was more common amongst men to communicate before the impending suicide attempt; It was primarily men who attempted suicide when under the influence of alcohol; The most common reason for attempting suicide was familial disharmony, including domestic violence. Financial issues and romantic conflicts were more common amongst males attempting suicide; A Glasgow Coma Scale (GCS) score of 9/15 and less at the time of presentation was associated with a higher mortality rate; Rodenticide consumption was the most lethal mode of attempted suicide, with a mortality rate of 66.7%.

As with the findings of our study, marriage appears to be less protective for Indian women than Indian men [8]. A hospital-based case-control study done in Kerala, India, to study the risk factors of suicide attempts in married women showed that intimate partner violence is a significant risk factor. Other risk factors included middle-income households, previous psychiatric history, and younger age group (<30 years) [9]. A study conducted in Chennai, India, in 1980 showed that married females below the age of thirty are predisposed to fatal suicidal behavior, thus necessitating the argument that this is a chronic trend that needs further analysis and intervention [10]. 

Poisoning was the most common method of attempted suicide in our study, followed by tablet overdose. A systematic review by Rane and Nadkarni reports hanging as the most frequent method (range 10%-72%) and poisoning (16%-49%) as the second most reported method of suicide [11]. Kumar et al. report poisoning as the most frequent method in males and females (31% and 48% respectively) [12]. The reason for the above could be ease of access to household poisons. Insecticides have been a preferred method in the Indian population over the years [11,13]. Self-immolation has a high female preponderance, with 63% of all self-immolations in India done by women in 2013 [14].

Rodenticide (usually, zinc/aluminium phosphide) poisoning has been found as the most lethal mode of suicide in our study. An open randomized study of 150 patients admitted in Haryana, India, for aluminum phosphide poisoning, a common constituent of rodenticides, saw a 77.2% mortality rate [15]. On the other hand, a study carried out in the state of Illinois, USA, comprising 37,352 hospital admissions for para-suicide and 10,287 completed suicides showed firearms to be the most lethal suicide method [16]. A similar trend was reported by Spicer and Miller in an evaluation of 10892 suicides and 57439 attempted suicides among hospital-admitted individuals across eight states in the United States [17].

The present study highlights the significant impact of financial and romantic conflicts on the mental health of men. A similar inference was drawn by an Indian research study that employed multiple correspondence analysis (MCA) to analyze National Crime Records Bureau (NCRB), India, data. It concluded that adult males succumb majorly to romantic relations, unemployment, and failure in examinations [18]. Financial and romantic strains were implied to be among the most common causes by Lal and Sethi in their study of 75 hospitalized patients in Lucknow, most of whom were males [19]. An Australian study of 262 suicide notes shows romantic failures in men to be the leading cause suggesting a lack of strong emotional support for men across different cultures [20]. 

Amongst all the men attempting suicide, 16.6% were under the influence of alcohol. In contrast, no women in our study had consumed alcohol at the time of the event. It may be due to the lack of females indulging in alcohol in rural India, a consistent finding across several studies in India owing to socio-cultural factors [13]. A survey by Bhattacharjee et al. showed similar results, with 17% of suicide attempters were under the influence of alcohol, predominantly male, particularly in the age group of 20-40. The use of alcohol was more in the illiterate and unskilled workers; however, high school educated persons and students used alcohol intentionally to facilitate suicide [21]. A meta-analysis done by Darvishi et al. concluded that alcohol use disorder was significantly associated with suicidal thoughts, attempts, and completed suicides [22]. 

A GCS score of 9/15 and below was associated with higher mortality in our study. Multiple studies on poisoning patients found significance and reliability in assessing GCS [23]. Cander et al. evaluated twenty-four acute organo-phosphorous poisoning cases and observed significantly lower GCS values in patients who died versus those who survived [24]. However, certain studies comparing GCS with the Full Outline of Unresponsiveness (FOUR) [25] and Acute Physiology and Chronic Health Evaluation Score (APACHE) II [26] scores argue that the latter two are better mortality indicators.

Limitations

The study was conducted while COVID-19 had been declared as a pandemic. Therefore, the financial, mental, and logistical impact of the pandemic may have influenced the social factors of suicide attempts. Also, the emotional mindset of the patients and attenders in the ER might have affected the PHQ-9 scores. The patients were not followed up after discharge.

## Conclusions

Attempted suicides form a sizeable number of patients presenting to the emergency room. Some interesting observations were made in the study regarding suicidal patterns in a rural area. Besides the presentation and GCS score, cognizance of the lethality of different methods in attempting suicide provides clues in anticipating the clinical course of the patient. The social patterns of suicide must be considered while designing awareness campaigns. Focused outreach programs must be conducted to high-risk groups such as married women, exam-going students, and alcoholic men to decrease the number of suicides. This study reinforces the importance of family therapy to promote a harmonious home environment. Strict policy decisions must be taken and enforced to limit the availability of household poisons.

## Figures and Tables

**Figure 6:**
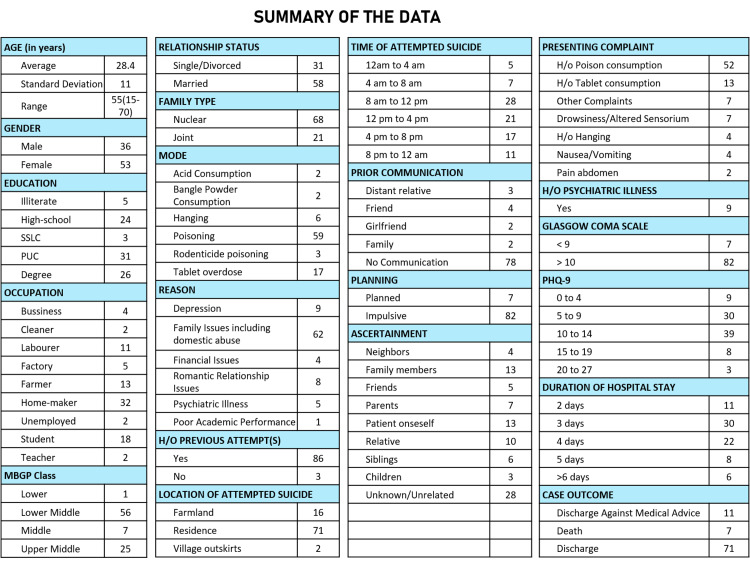
Summary of the data collected

## References

[1] Thippaiah SM, Nanjappa MS, Math SB (2019). Suicide in India: a preventable epidemic. Indian J Med Res.

[2] Buzan RD, Weissberg MP (1992). Suicide: risk factors and therapeutic considerations in the emergency department. J Emerg Med.

[3] Pompili M, Innamorati M, Serafini G (2011). Suicide attempters in the emergency department before hospitalization in a psychiatric ward. Perspect Psychiatr Care.

[4] (2018). Gender differentials and state variations in suicide deaths in India: the Global Burden of Disease Study 1990-2016. Lancet Public Health.

[5] Rives W (1999). Emergency department assessment of suicidal patients. Psychiatr Clin North Am.

[6] Betz ME, Boudreaux ED (2016). Managing suicidal patients in the emergency department. Ann Emerg Med.

[7] Weber AN, Michail M, Thompson A, Fiedorowicz JG (2017). Psychiatric emergencies: assessing and managing suicidal ideation. Med Clin North Am.

[8] Vijayakumar L (2015). Suicide in women. Indian J Psychiatry.

[9] Indu PV, Remadevi S, Vidhukumar K, Shah Navas PM, Anilkumar TV, Subha N (2020). Domestic violence as a risk factor for attempted suicide in married women. J Interpers Violence.

[10] Ponnudurai R, Jeyakar J (1980). Suicide in Madras. Indian J Psychiatry.

[11] Rane A, Nadkarni A (2014). Suicide in India: a systematic review. Shanghai Arch Psychiatry.

[12] Kumar S, Verma AK, Bhattacharya S, Rathore S (2013). Trends in rates and methods of suicide in India. Egyptian Journal of Forensic Sciences, Volume.

[13] Adityanjee DR (1986). Suicide attempts and suicides in India: cross-cultural aspects. Int J Soc Psychiatry.

[14] (2020). Crime in India Year 2013. https://ncrb.gov.in/en/crime-india-year-2013.

[15] Chugh SN, Kumar P, Aggarwal HK, Sharma A, Mahajan SK, Malhotra KC (1994). Efficacy of magnesium sulphate in aluminium phosphide poisoning--comparison of two different dose schedules. J Assoc Physicians India.

[16] Shenassa ED, Catlin SN, Buka SL (2003). Lethality of firearms relative to other suicide methods: a population based study. J Epidemiol Community Health.

[17] Spicer RS, Miller TR (2000). Suicide acts in 8 states: incidence and case fatality rates by demographics and method. Am J Public Health.

[18] Kamalja KK, Khangar NV (2017). A statistical study of suicidal behavior of Indians. Egypt J Forensic Sci.

[19] Lal N, Sethi BB (1975). Demographic and socio-economic variables in attempted suicide by poisoning. Indian J Psychiatry.

[20] Lester D, Wood P, Williams C, Haines J (2021). Motives for suicide—a study of Australian suicide notes. Crisis.

[21] Bhattacharjee S, Bhattacharya A, Thakurta RG, Ray P, Singh OP, Sen S (2012). Putative effect of alcohol on suicide attempters: an evaluative study in a tertiary medical college. Indian J Psychol Med.

[22] Darvishi N, Farhadi M, Haghtalab T, Poorolajal J (2020). Alcohol-related risk of suicidal ideation, suicide attempt, and completed suicide: a meta-analysis. PLoS One.

[23] Heard K, Bebarta VS (2004). Reliability of the Glasgow Coma Scale for the emergency department evaluation of poisoned patients. Hum Exp Toxicol.

[24] Cander B, Dur A, Yildiz M, Koyuncu F, Girisgin AS, Gul M, Okumus M (2011). The prognostic value of the Glasgow coma scale, serum acetylcholinesterase and leukocyte levels in acute organophosphorus poisoning. Ann Saudi Med.

[25] Jalali R, Rezaei M (2014). A comparison of the glasgow coma scale score with full outline of unresponsiveness scale to predict patients' traumatic brain injury outcomes in intensive care units. Crit Care Res Pract.

[26] Zali AR, Seddighi AS, Seddighi A, Ashrafi F (2012). Comparison of the acute physiology and chronic health evaluation score (APACHE) II with GCS in predicting hospital mortality of neurosurgical intensive care unit patients. Glob J Health Sci.

